# Epidemiology, Antimicrobial Resistance, and Virulence Determinants of Group B *Streptococcus* in an Australian Setting

**DOI:** 10.3389/fmicb.2022.839079

**Published:** 2022-06-14

**Authors:** Sandra Jones, Peter Newton, Matthew Payne, Lucy Furfaro

**Affiliations:** ^1^Microbiology, NSW Health Pathology, Wollongong Hospital, Wollongong, NSW, Australia; ^2^Graduate Medicine, University of Wollongong, Wollongong, NSW, Australia; ^3^Division of Obstetrics and Gynaecology, School of Medicine, The University of Western Australia, Perth, WA, Australia; ^4^Women and Infants Research Foundation, Subiaco, WA, Australia

**Keywords:** group B *Streptococcus*, *Streptococcus agalactiae*, whole genome sequencing, antimicrobial resistance, epidemiology, serotyping, surveillance

## Abstract

*Streptococcus agalactiae* [group B *Streptococcus* (GBS)] is a major neonatal pathogen and also causes invasive disease in non-pregnant adults. One hundred GBS isolates (*n* = 50 invasive disease and *n* = 50 colonizing pregnant women) were characterized using capsular serotyping by latex agglutination, antimicrobial susceptibility testing, and whole genome sequencing (WGS). All isolates were susceptible to penicillin, 32% were resistant to clindamycin. Of these, two isolates had reduced susceptibility to ceftriaxone (MIC 0.75 mg/L) and were found to have unique alleles at *pbp2X* and *pbp1A*. Capsular serotypes Ia (18%), III (18%), Ib (14%), V (12%), and VI (11%) were most common and comparison of latex agglutination and capsular genotyping by WGS showed 71% agreement. Less common capsular genotypes VI–VIII represented 15% of isolates, indicating that a significant proportion may not be targeted by the proposed pentavalent or hexavalent vaccines under development. WGS is a useful aid in GBS surveillance and shows correlation to phenotypic serotyping and antimicrobial susceptibility data.

## Introduction

*Streptococcus agalactiae* [group B *Streptococcus* (GBS)] has been recognized as a major neonatal pathogen since the 1970s (Wilkinson, 1978) and has also emerged as an increasing cause of invasive infections in non-pregnant adults in recent decades (Farley, 2001; Phares et al., 2008). The epidemiology of GBS colonization and disease is dynamic, but gaps remain in our understanding of the species without robust surveillance systems in place. Characterization of isolates circulating within local populations provides critical information regarding trends in antimicrobial resistance, helping to inform local antibiotic treatment, and prevention strategies, and also helps guide the implementation of alternative approaches, such as vaccines and novel therapeutic options.

In neonates, GBS causes early-onset (sepsis and pneumonia within the first 7 days of life) and late-onset disease (meningitis and sepsis between 7 days and 3 months of age) (Wilkinson, 1978). The greatest risk factor is maternal colonization, and rates vary according to geography [for example, rates of ~12% in South and East Asian countries, compared to ~35% in the Caribbean (Russell et al., 2017a)], ethnicity, sexual activity, and socioeconomic status (Furfaro et al., 2019). In Australia, maternal colonization rates of 19–27% have been reported (Gilbert et al., 2002; Hiller et al., 2005; Furfaro et al., 2019). A substantial decline in early-onset disease was seen in the US (Centers for Disease Control Prevention., 2010), Australia, (Isaacs and Royle, 1999) and globally (Russell et al., 2017b) following implementation of the US Centers for Disease Control and Prevention guidelines for use of intrapartum antimicrobial prophylaxis (IAP) in 1996 (Centers for Disease Control Prevention., 1996), and since then, both culture-based and risk-based approaches (Table 1) have been in widespread use. IAP regimes utilize intravenous penicillin, cefazolin in non-severe penicillin hypersensitivity, and clindamycin or vancomycin in severe penicillin hypersensitivity (Antibiotic version 16, 2019). However, effective implementation requires coordination between multiple healthcare providers and administration of antimicrobials at least 4 h prior to delivery, which continues to face barriers in real-world practice (Le Doare et al., 2017; Nanduri et al., 2019). In addition, long-held concerns regarding the effect of IAP on the emergence of antimicrobial resistance and on the infant microbiome (Seedat et al., 2017), have led to a shift in research interest to alternative prevention strategies, such as a maternal GBS vaccine, which may also reduce late-onset disease. Invasive GBS infections in non-pregnant adults most often occurs in patients with underlying risk factors such as diabetes mellitus, cardiovascular disease, liver disease, chronic kidney disease, obesity, malignancy, and age over 65 years (Bennett et al., 2015; Francois Watkins et al., 2019). A wide spectrum of disease occurs, such as primary bacteremia, skin and soft tissue infections, pneumonia, meningitis, endocarditis, septic arthritis, and osteomyelitis, and it is proposed that this population may also benefit from a GBS vaccine in the future (Farley, 2001).

**Table 1:** Indications for intrapartum antibiotic prophylaxis (IAP) (see Footnote 1; Centers for Disease Control Prevention., 2010).

History of invasive GBS infection in a neonate from a previous pregnancy	
GBS bacteriuria during current pregnancy	
Risk-based	- Intrapartum fever (≥ 38.0°C) - Preterm onset of labor (<37 weeks) - Prolonged rupture of membranes (≥ 18 h)
Culture-based	- GBS cultured from vaginal and rectal swabs at 36 0/7 to 37 6/7 weeks' gestation

Although GBS has remained susceptible to penicillins and first-generation cephalosporins in the face of widespread IAP use, isolates, with increasing penicillin minimum inhibitory concentrations (MICs) have recently been reported in Japan (Kimura et al., 2008; Nagano et al., 2008), the US (Dahesh et al., 2008; Metcalf et al., 2017) and Canada (Gaudreau et al., 2010; Longtin et al., 2011), posing a threat to the advances made in disease prevention and treatment. Resistance to second-line antibiotics such as erythromycin and clindamycin varies widely, with rates up to 74.1% in China (Lu et al., 2016) and 65.9% in Taiwan (Kao et al., 2019), limiting treatment options for patients with severe penicillin hypersensitivity. Investigation into GBS virulence factors informs our understanding of disease processes and may also help guide the development of novel prevention or therapeutic strategies. Several have been identified in animal models and *in vitro* studies (Lindahl et al., 2005; Vornhagen et al., 2017) including the polysaccharide capsule, CAMP factor, secreted hemolysins and laminin-binding protein (Lmb), which is involved in cell adhesion. Other proteins hypothesized to play a role in disease include Rib surface protein (Stalhammar-Carlemalm et al., 1993), C5a peptidase (a serine protease encoded by *scpB*, that interferes with neutrophil recruitment) (Lindahl et al., 2005) and hyaluronidase (which promotes vaginal colonization) (Vornhagen et al., 2017).

Historically, serotyping based on capsular polysaccharide antigenic differences has been used, with 10 different serotypes characterized (Ia, Ib, II, III, IV, V, VI, VII, VIII, and IX), and a small proportion described as a non-typable (NT). In the 1970's and 80's, type III strains were predominant in neonatal sepsis, before the emergence of Ia and V in the 1990's. Most recent data on neonatal sepsis in the US (between 2006 and 2015) reports Ia, Ib, II, III, IV, and V cause >99% cases of early-onset and late-onset disease (Nanduri et al., 2019), with similar findings found in a meta-analysis of global neonatal sepsis data (Madrid et al., 2017). Serotypes Ia, Ib, II, III, and V also predominate in invasive isolates from non-pregnant adults in the US, causing >86% cases (Phares et al., 2008; Francois Watkins et al., 2019), and serotype IV has also recently significantly increased (Francois Watkins et al., 2019). Available data on invasive isolates in Australia also show a predominance of serotype Ia, III and V (Zhao et al., 2008; Stewart et al., 2020). A number of vaccines have undergone clinical trials (Baker et al., 2003, 2004), and it is predicted that a pentavalent conjugate vaccine (including types Ia, Ib, II, III, V) could potentially prevent up to 96% of neonatal disease and 88% of adult disease in the US (Phares et al., 2008). Multi-locus sequence typing (MLST) is another method based on typing of seven defined housekeeping genes (*adhP, atr, glcK, glnA, pheS, sdhA*, and *tkt*) to produce related sequence types (ST) (Jones et al., 2003). In neonatal sepsis studies, ST23, ST22 and ST17 are the most common causing early-onset disease, and ST17, ST23, and ST19 the most common causing late-onset disease (Nanduri et al., 2019). ST17 is thought to be highly virulent, showing the strongest correlation with invasive neonatal disease and meningitis (Martins et al., 2007). By comparison, there is limited data regarding ST epidemiology in non-pregnant adults.

This study aimed to characterize GBS isolates from the Illawarra Shoalhaven region in NSW, Australia, utilizing antimicrobial susceptibility testing, serotyping, and whole genome sequencing (WGS), a method that may potentially improve our understanding of the epidemiology, pathogenesis, and resistance determinants of the organism.

## Article Types–Original Research Article

### Methods

#### Isolate Collection

Ethics approval was obtained through the Joint University of Wollongong and Illawarra Shoalhaven Local Health District Health and Medical Human Research Ethics Executive Committee (2019/ETH00652). One hundred isolates of GBS were collected from clinical samples from the Illawarra Shoalhaven Local Health District, south of Sydney NSW, between March 2016 and March 2019. Fifty isolates were obtained from rectovaginal screening swabs collected during pregnancy, and the remaining 50 were obtained from blood cultures (*n* = 44), synovial fluid (*n* = 2), tissue culture (*n* = 1), wound swabs (*n* = 2), and placental swab (*n* = 1). Identification was confirmed using MALDI-TOF MS Biotyper® (Bruker Daltronics, Germany) and Lancefield grouping by slide agglutination (Oxoid^TM^ Streptococcal Grouping Kit, Thermo Fisher Scientific). Isolates were preserved in sterile nutrient broth with 15% glycerol at −80°C until analysis.

#### Antimicrobial Susceptibility Testing

Antimicrobial susceptibility testing was performed using Vitek® 2 AST-ST03 (bioMérieux, Marcy l'Etoile, France), and minimum inhibitory concentrations (MICs) interpreted based on EUCAST guidelines (Table 2). Results were compared to previous agar disc diffusion testing that had been conducted using the Calibrated Dichotomous Susceptibility (CDS) method (Bell et al., 2019). Variables between different groups were analyzed using an online calculator socscistatistics.com (Z-score calculator for two population proportions), with a two-tailed *p*-value of < 0.05 being considered significant.

**Table 2:** EUCAST MIC breakpoints for Streptococcus groups A, B, C and G (mg/L).

	**S ≤**	**R >**
Benzylpenicillin	0.25	0.25
Ceftriaxone	Note^1^	Note^1^
Vancomycin	2	2
Erythromycin	0.25	0.5
Clindamycin	0.5	0.5
Tetracycline	1	2
Moxifloxacin	0.5	0.5
Levofloxacin	0.001	2
Trimethoprim-sulfamethoxazole	1	2
Chloramphenicol	8	8

*Note^1^: the susceptibility to cephalosporins is inferred from the benzylpenicillin susceptibility*.

#### Capsular Serotyping

Capsular serotyping of each isolate was performed by the latex agglutination method with specific antisera against types Ia to IX capsular polysaccharide antigens (Statens Serum Institute, SSI Diagnostica, Denmark), after overnight culture in Todd Hewitt Broth with Colistin and Nalidixic Acid (ThermoScientific).

#### DNA Extraction

DNA was extracted from each isolate (and negative controls) using the MagAttract® Microbial DNA Kit (QIAGEN) on an automated Kingfisher Flex 96 magnetic particle processor, after incubating single colonies of each in tryptic soy broth for 24 h at 35°C. Bacterial DNA was fragmented using the Ion Xpress^TM^ Plus Fragment Library Kit (ThermoFisher Scientific), unique barcode adapters ligated and the resulting barcoded library amplified at the manufacturers specified PCR cycling conditions before library purification using Agencourt^TM^ AMPure^TM^ XP bead-based size selection. The amplified library (100 pM) was templated and sequenced using the Ion 520 & Ion 530 ExT Kit and Ion 530 Chip on the Ion Chef instrument and Ion S5 System (Thermo Fisher).

#### Sequencing

Data analysis was performed by importing raw reads into Geneious (version 10.2.4) bioinformatics platform, and assembly using SPAdes (version 3.10.0). The resulting assembly FASTA files were uploaded to QUAST (version 4.6) and analyzed for genomic quality (Gurevich et al., 2013). Assembly FASTA files were also uploaded to the *S. agalactiae* PubMLST database (https://pubmlst.org/sagalactiae/) and curated using the BIGSdb (Jolley and Maiden, 2010) to obtain MLST sequence types (ST) and annotations for genes of interest. Capsular genotypes were determined using reference sequences for capsular serotypes previously published (Sheppard et al., 2016) and these were mapped to contigs for each isolate to generate the pairwise identity percentage score to determine the most probable genotype. Additionally, *in silico* analyses of primers (Imperi et al., 2010) for all 10 genotypes were used to confirm capsular genotypes, and raw reads were analyzed using GBS-SBG (GBS Serotyping by Genome Sequencing; Tiruvayipati et al., 2021). Assembled contigs were assessed for antimicrobial resistance genes (Appendix 1) *via* ABRicate (https://github.com/tseemann/abricate) using databases including NCBI (https://www.ncbi.nlm.nih.gov/gene/) (Gene. Bethesda (MD): National Library of Medicine (US), 2004) (Appendix 1), ResFinder (version 4.1; Bortolaia et al., 2020) and CARD (The Comprehensive Antibiotic Resistance Database platform 1.0; Alcock et al., 2020). Similarly, genes hypothesized to be associated with GBS virulence were also obtained through NCBI and virulence factor database (VFDB) (Appendix 1; Chen et al., 2016).

## Results

### Patient Characteristics

A total of 50 GBS isolates from rectovaginal screening swabs were obtained from 50 pregnant women with a mean age of 29.9 years (range 18–42 years). An additional 50 isolates causing invasive disease were obtained from 50 patients with a mean age of 71.9 years (range first day of life to 95 years) and a female predominance of 60%. All patients with invasive disease had at least one of the recognized risk factors. The clinical syndromes and specimen types for invasive isolates are represented in Table 3. Out of these, 11 blood culture isolates were deemed to be of unclear source either after appropriate investigation, or due to limited assessment having been conducted.

**Table 3:** Specimen types for invasive GBS isolates included in the study (*n* = 50).

***Clinical syndrome***	**Blood culture**	**Synovial fluid**	**Tissue**	**Swab**
Infective endocarditis	2			
Skin and soft tissue infection	12		1	
Septic arthritis / prosthetic joint infection	6	2		
Osteomyelitis	2			2
Diabetic foot infection	6			
Pneumonia	2			
Dental infection	1			
Bowel carcinoma	1			
Neonatal early onset disease	1			
Stillbirth				1
Bacteraemia of unclear source	11			

### Antimicrobial Susceptibility

The antibiotic susceptibility profiles obtained using Vitek® 2 AST-ST03 are shown in Table 4. Overall there was a 99% correlation to the results obtained using the CDS method. One very major error was identified in an isolate that appeared tetracycline susceptible on CDS testing but resistant using Vitek and was found to harbor the *tetM* gene on whole genome sequencing. Three isolates were determined to have the constitutive MLS_B_ phenotype by Vitek but had tested erythromycin susceptible on CDS testing. Three isolates had inducible clindamycin resistance not detected by CDS testing, and one isolate had a constitutive MLS_B_ phenotype, which appeared to be inducible on CDS testing. Two invasive isolates (WOL-1-10 and WOL-1-18) had reduced susceptibility to ceftriaxone on both CDS and Vitek methods, and on further testing using E-test (bioMérieux) had penicillin MICs of 0.023 mg/L and 0.025 mg/L, and ceftriaxone MICs of 0.75 mg/L for each.

**Table 4:** Phenotypic antibiotic susceptibility profiles of GBS isolates, as tested by Vitek® 2 AST-ST03.

**Antibiotic**	**Colonizing (*n* = 50)**	**Invasive (*n* = 50)**
Penicillin	100%	100%
Ceftriaxone	100%	96%
Vancomycin	100%	100%
Erythromycin	68%	68%
Clindamycin	68%	68%
- constitutive MLS_B_ phenotype	12%	18%
- inducible clindamycin resistance	20%	14%
Tetracycline	14%	32% (*p = 0.03)*
Moxifloxacin	100%	98%
Levofloxacin	96%	96%
Trimethoprim-sulfamethoxazole	100%	100%
Chloramphenicol	100%	100%

### Serotyping by Latex Agglutination

**Figure 1:**
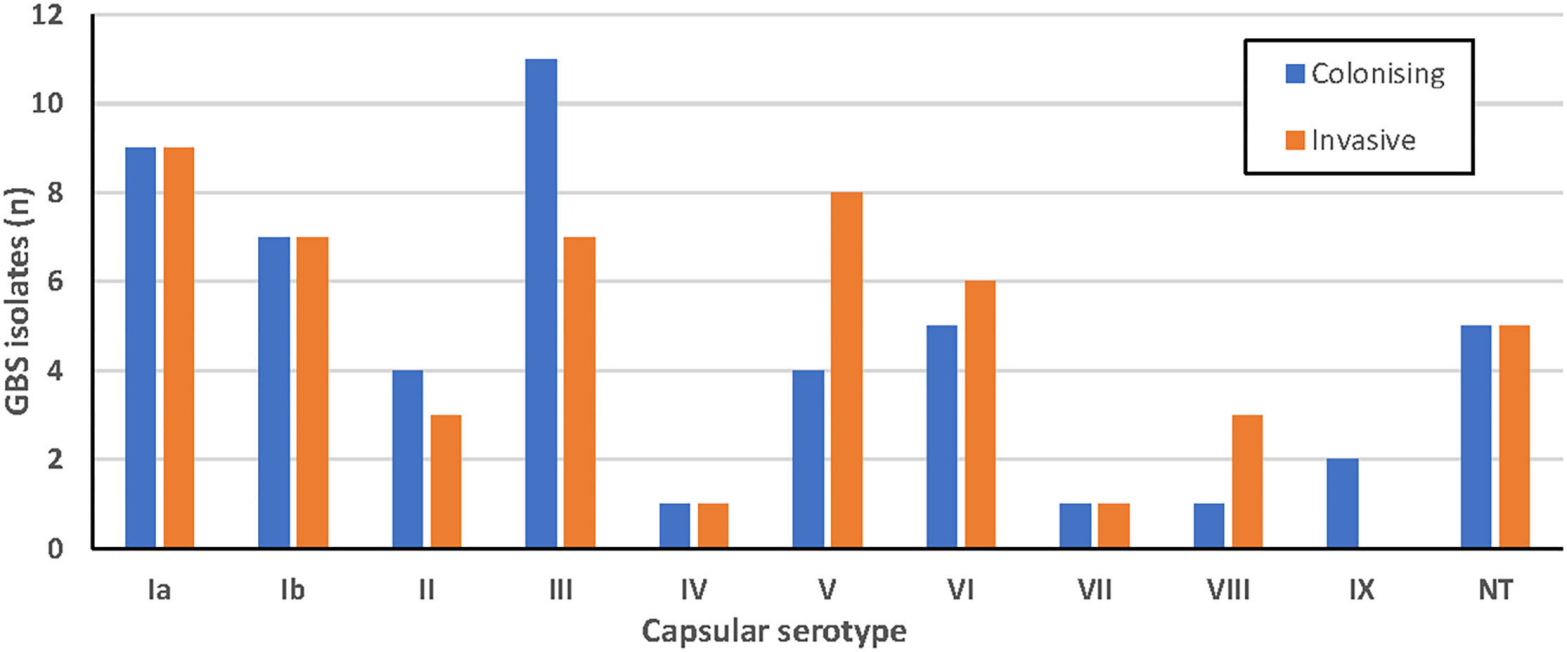
Serotypes of colonizing (blue; *n* = 50) and invasive (orange; *n* = 50) GBS isolates obtained using latex agglutination.

GBS serotypes as determined by latex agglutination are shown in Figure 1. Results were determined according to the strongest agglutination reaction observed, although a milder agglutination with a second serotype was also seen in 6% of isolates. Ten isolates did not produce any agglutination reaction with any antisera and were categorized as non-typable (NT). There was no apparent association between serotype and clinical syndrome for the invasive isolates (Appendix 2).

### Whole Genome Sequencing Data

**Figure 2:**
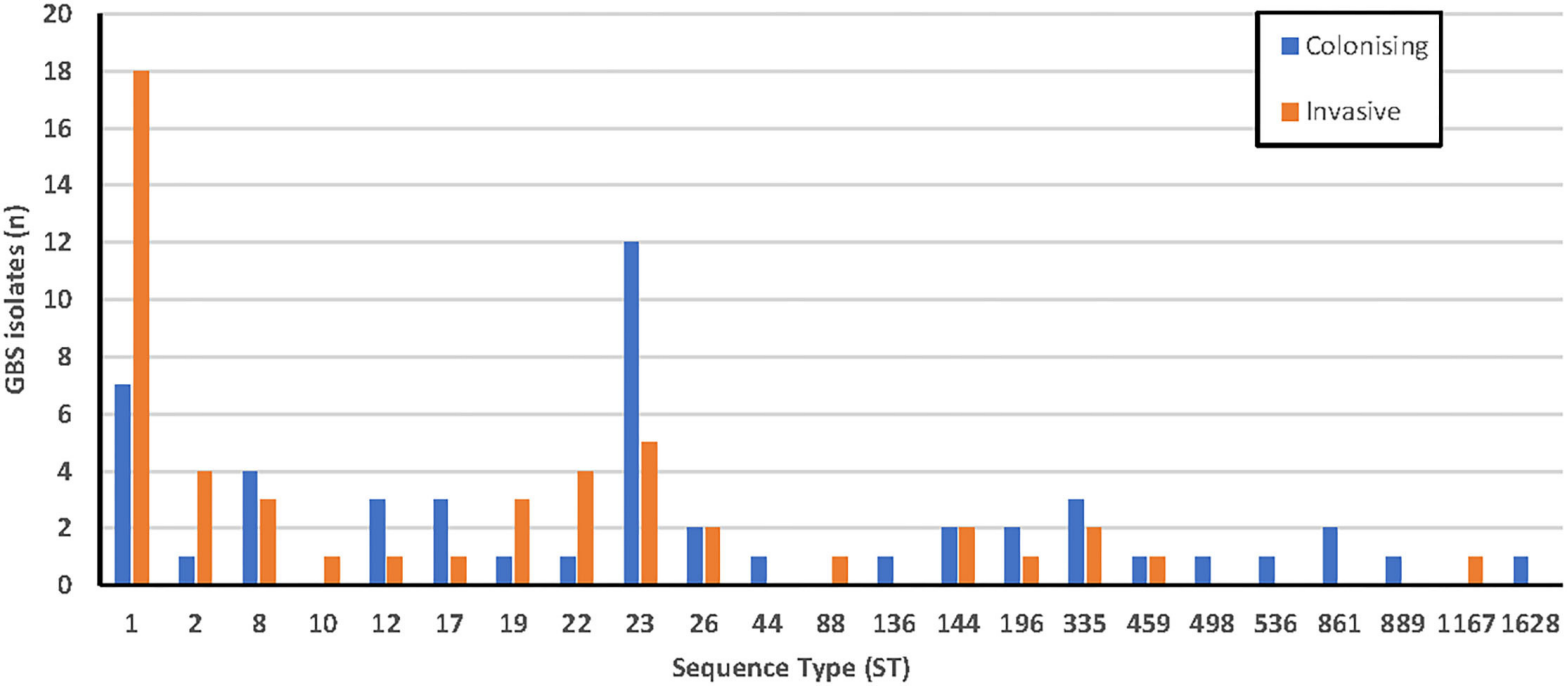
Sequence types of colonizing (*n* = 50) and invasive (*n* = 50) GBS isolates using MLST.

Assembled data of sufficient quality were obtained from 100 isolates (Appendix 3). After curation on the PubMLST database, sequence types (ST) were obtained for each, shown in Figure 2. A ratio of 2.6:1 invasive:colonizing isolates was observed for ST1. In contrast, ST23 isolates were predominantly colonizing isolates (colonizing: invasive ratio of 2.4:1). There did not appear to be a correlation between sequence type and clinical syndrome for the invasive isolates (Appendix 4).

Comparison of capsular serotypes by latex agglutination and genotypes by WGS analyses showed a 71% agreement between the two methods (Table 5). All NT isolates by latex agglutination (*n* = 10) were able to be categorized into a capsular genotype including Ia (*n* = 5), II (*n* = 2), IV (*n* = 2), and V (*n* = 1). Interestingly, one isolate (WOL-2-22) returned positive agglutination for both V and VI capsular serotypes. This was also confirmed by genotyping as the isolate contained both CpsN and CpsI targets from *in silico* primer testing (Imperi et al., 2010). Within each sequence type, an association with certain genotypes obtained *via* WGS was observed (Table 6).

**Table 5:** Comparison of capsular genotypes of sequenced study isolates (*n* = 100) compared to capsular serotype obtained by latex agglutination.

	**Capsular genotyping (** ***n*** **)**											
**Latex agglutination (*n*)**	**Ia**	**Ib**	**II**	**III**	**IV**	**V**	**VI**	**VII**	**VIII**	**IX**	**NT**	**Total**
Ia	14					4						18
Ib		8	2		3^∧^		1					14
II			7									7
III				17		1						18
IV					1	1						2
V		1	1			9						11
VI	1			1			9					11
VII					1			1				2
VIII									4			4
IX	1	1										2
NT	5		2		2	1						10
Total	21	10	12	18	7	16	10	1	4	0	0	99^*^

* *One isolate excluded from the table V/VI by both latex and genotyping. ^∧^One isolate with Ib positive/weak IV agglutination with genotyping as cps IV*.

**Table 6:** Association of MLST sequence types (ST) to capsular genotype.

**ST**	**Capsular genotype**
1 (*n* = 25)	V (11/25), VI (9/25), Ia (4/25), VII (1/25)
2 (*n* = 5)	VIII (4/5), IV (1/5)
8 (*n* = 7)	Ib (7/7)
12 (*n* = 4)	II (3/4), Ib (1/4)
17 (*n* = 4)	III (4/4)
19 (*n* = 4)	III (4/4)
22 (*n* = 5)	II (5/5)
23 (*n* = 17)	Ia (16/17), III (1/17)
26 (*n* = 4)	V (4/4)
144 (*n* = 4)	Ia (4/4)
196 (*n* = 3)	IV (3/3)
335 (*n* = 5)	III (5/5)
459 (*n* = 2)	IV (2/2)
861 (*n* = 2)	III (2/2)

All isolates were phenotypically susceptible to penicillin, and all showed ≥99.0% pairwise alignment to the beta-lactam-related *pbp1A, pbp2A, pbp1B, pbp2B, pbp2X, fibA*, and *fibB* genes, except one isolate that did not match to *pbp1A*. The two isolates (WOL-1-10 and WOL-1-18) with reduced susceptibility to ceftriaxone harbored unique alleles for *pbp2X* (allele 344) and *pbp1A* (allele 353) not observed for any other isolate on the PubMLST database. All isolates appeared susceptible to trimethoprim/sulfamethoxazole and chloramphenicol on phenotypic testing, and all showed ≥99.80% pairwise alignment to *folA* and ≥96.90% alignment to *cat*. One isolate (WOL-1-16) was phenotypically resistant to levofloxacin and moxifloxacin with MICs of ≥16 and ≥4, respectively. Analyses of the quinolone-resistance genes detected substitutions in both *parC* (S79F) and *gyrA* (S81L) genes. Additionally, three other isolates resistant to levofloxacin only (MIC = 4 mg/L) were found to harbor *parC* substitutions including S79F (WOL-1-77) and D83N (WOL-1-2 and WOL-1-37). A total of 77% isolates were phenotypically resistant to tetracycline, with the *tetM* gene found in the majority (63/77) and *tetO* gene found in most of the remainder (12/77). *tetM* and *tetO* were both only found in one susceptible isolate each.

Thirty two percent of isolates were phenotypically resistant to both erythromycin and clindamycin (15 of constitutive MLS_B_ and 17 of inducible MLS_B_ phenotype), while the remaining 68% were susceptible to both. *ermB* was detected in the majority of constitutive phenotypes (10/15), followed by *lsa* (4/15), while *ermA* was detected in the majority of inducible phenotypes (11/17), followed by *mefA* + *mel* + *msr (D)* (3/17) and *erm T* (2/17). These results are summarized in Table 7.

**Table 7:** Comparison of presence of macrolide resistance genes to phenotypic susceptibility testing for sequenced isolates (*n* = 100).

**Phenotype**	***mefA* + *mel* + *msr (D)***	***ermA***	***ermB***	***ermT***	***lsa***
Constitutive MLS_B_ (*n* = 15)	1	1	10	0	4
Inducible MLS_B_ (*n* = 17)	3	11	1	2	0
Erythromycin and clindamycin susceptible (*n* = 68)	0	1	3	0	2

The virulence genes *cfb, cylE, lmb, scpB* and *pavA* were found in ≥99% isolates, and *rib* was not detected in any isolate. *fbp* was found in 63% isolates (34 invasive, 29 colonizing) with pairwise alignment ≥99.8%. *hylB* was found in 86% isolates (not found in six invasive, eight colonizing isolates).

## Discussion

Serotypes Ia, III and V were most common in this study using both latex agglutination and whole genome sequencing. Capsular genotype Ia was predominant in colonizing strains (*n* = 14) and capsular genotype V was the most common invasive strain (*n* = 12), and has also been reported to be associated with invasive disease in the US and Canada (Teatero et al., 2017). The capsular genotypes of 71% were in agreement with latex agglutination serotypes, suggesting that typing done by WGS can be correlated to some degree to historical latex agglutination data published in the literature. However, both methods have their limitations to consider if using either alone. While capsular genotyping had the advantage of being able to assign the entire 10 NT isolates a genotype, information about the function and expression of these genes is not clear. While rapid, latex agglutination testing is limited by the subjective observation of agglutination, which may lead to interobserver error.

In contrast to the significant predominance of serotypes Ia, Ib, II, III, IV and V in neonatal sepsis and invasive disease in non-pregnant adults in the US, the isolates from the Illawarra Shoalhaven region showed more heterogeneity with 15% comprised of capsular genotypes VI-VIII using WGS, which have been reported to be more common in Asia (Lachenauer et al., 1999; Eskandarian et al., 2015; Russell et al., 2017a). The proposed pentavalent conjugate vaccine (including types Ia, Ib, II, III, V) would not cover these or serotype IV, leaving 22% of the studied isolates not included in prevention measures. More recently, a hexavalent vaccine (Ia, Ib, II, III, IV and V) has been under development (Buurman et al., 2019), but this would still leave 15% of the studied isolates without coverage. Including serotype VI in vaccine targets would significantly reduce those without vaccine coverage to only 5% of studied isolates in our region; however it would be important to continue to monitor surveillance of serotypes VII–IX over time.

Characterization of isolates by MLST found that ST1 (25%) and ST23 (17%) were the most common, with ST1 predominating in invasive infections (36%) and ST23 predominating colonizing isolates (24%). This is consistent with Australian data (176 isolates) available on the *S. agalactiae* PubMLST database, with ST19, ST17, and ST12 also being common, and also with a recent study of 32 invasive isolates from the Sunshine Coast region in Queensland, Australia (Stewart et al., 2020). There was correlation noted between certain STs and serotypes (ST1 with serotypes V and VI, ST23 with serotype Ia, ST17, and ST19 with serotype III, ST8 with serotype Ib, and ST12 with serotype II) which have all been reported previously (Furfaro et al., 2018). Less commonly reported STs found in this study include ST22, ST2, ST335, ST26, and ST144. ST17 is often reported in the literature as being highly associated with invasive neonatal disease and meningitis (Luan et al., 2005; Lin et al., 2006; Martins et al., 2007), however only four isolates were found in this study, three of which were colonizers.

Despite the widespread use of IAP since the 1990's, GBS remains susceptible to penicillin and first-generation cephalosporins (Nanduri et al., 2019), a finding which was confirmed in this study. However, isolates with increasing MICs of 0.25–1.0 mg/L for penicillin have been reported in Japan (Kimura et al., 2008; Nagano et al., 2008), the US (Dahesh et al., 2008; Metcalf et al., 2017) and Canada (Gaudreau et al., 2010; Longtin et al., 2011) mediated *via* alterations in the penicillin-binding protein genes *pbp1A, pbp2A, pbp2B*, and *pbp2X*. Two invasive isolates in this study were found to have reduced susceptibility to ceftriaxone while remaining susceptible to penicillin, a phenotype also previously reported in Iran (Jannati et al., 2012), and unique alleles were identified for *pbp1A* and *pbp2X*. Although this does not impact current antibiotic treatment and IAP guidelines, it does further inform our understanding of the role of penicillin-binding proteins in beta-lactam therapy for this organism. Conversely, tetracycline resistance is common in GBS with rates increasing over time to nearly 90% in some areas (Metcalf et al., 2017; Teatero et al., 2017), with 77% seen in this study. Resistance was significantly higher in colonizing strains compared to those causing invasive disease (86 vs. 68%; *p* = 0.03), and most commonly mediated by the presence of the *tetM* gene (63/77) or *tetO* gene (12/77), encoding ribosomal protection proteins.

Macrolide and lincosamide resistance in GBS poses a threat to disease treatment and prevention in patients with severe penicillin hypersensitivity, with an estimated 5–15% of patients in developed countries carrying a penicillin allergy label (Blumenthal et al., 2019). The majority presents as an MLS_B_ phenotype, either constitutively expressed or inducible, due to the presence of *erm* genes (*ermA, ermB, ermT)* encoding erythromycin ribosomal methylase which modifies the 23S rRNA target-binding site resulting in co-resistance to macrolides, lincosamides and streptogramins B. Resistance to both erythromycin and clindamycin was found in 32% of all isolates in this study, which is similar to recently reported rates in the US (Nanduri et al., 2019) and Canada (De Azavedo et al., 2001; Teatero et al., 2017), but higher than that reported in Asia (Eskandarian et al., 2015). *ermA* and *ermT* were associated with inducible MLSB phenotype (13/17 isolates) in this study, and *ermB* with constitutive phenotype (10/15 isolates). *lsa*, encoding an efflux pump, was found in most of the remaining constitutively resistant phenotypes (4/15), and a combination of *mefA* + *mel* + *msr(D)* (also encoding efflux pumps) found in most of the remaining inducible phenotypes (3/17). *mel* has previously been reported to be associated with *mefA* in GBS (Marimón et al., 2005). An association between macrolide resistance and serotype V has previously been reported (Zeng et al., 2006; Phares et al., 2008; Nanduri et al., 2019), and this was seen in this study with 10 of the 32 resistant isolates aligning to the reference sequence for serotype V (and 10 of the 14 isolates aligning to serotype V reference sequence phenotypically resistant).

Quinolone resistance may be mediated *via* mutations in *gyrA, gyrB, parC*, and *parE*, which modify the binding sites on topoisomerases and gyrases involved in DNA replication. All fluoroquinolone-resistant isolates (*n* = 53) from a recent whole genome sequencing study of invasive GBS isolates in the US were found to harbor substitutions in *parC* and/or *gyrA* genes (Metcalf et al., 2017). Only one fluoroquinolone-resistant isolate (WOL-1-16) was observed in this study, and was found to contain the same *parC* and *gyrA* substitutions described previously (Metcalf et al., 2017). Additionally, the other isolates resistant to levofloxacin only were found to harbor only *parC* substitutions, consistent with sequential stepwise mutations conferring resistance to newer-generation fluoroquinolones.

The virulence genes *cfb, lmb, pavA, cylE*, and *scpB* were present in almost all isolates, which is consistent with other studies (Eskandarian et al., 2015). *rib* was not found in any of the sequenced isolates in this study but has been found at rates of 20–57% in other reports (Manning et al., 2006; Lin et al., 2009; Eskandarian et al., 2015), and has been associated with serotype II and III strains (Lindahl et al., 2005; Eskandarian et al., 2015). Interestingly, *fbp* was only found in 68% invasive isolates and 58% colonizing isolates, and encodes fibronectin-binding protein, which may play a role in endothelial adhesion. Also, *hylB*, encoding hyaluronidase that is thought to play a role in colonization, was not found in 14% isolates (six invasive and eight colonizers) in this study. Further investigation is required to elucidate the contribution that these genes, and many others under investigation, provide in colonization and organism invasion, and also host factors influencing susceptibility to disease.

This study has certain limitations that should be acknowledged. Without a comprehensive surveillance system in place, the samples were limited to 100 select isolates, with minimal representation from neonatal sepsis, so are not representative of the local population structure. In addition, clinical patient data was limited, so application of these findings to treatment outcomes and mortality are not possible.

In conclusion, epidemiological characterization of GBS strains is useful to gain insights into mechanisms of disease, monitor antimicrobial resistance and to find common features among strains that enable us to broadly target GBS in terms of prevention and treatment. Higher proportions of capsular genotypes VI–VIII than expected were found in the Illawarra Shoalhaven region, raising the possibility that a pentavalent or hexavalent vaccine may provide less coverage than predicted elsewhere. The rates of macrolide and clindamycin resistance seen in this study and described elsewhere indicate that clindamycin is not a reliable empirical treatment or prophylaxis option, and highlights the need for antimicrobial susceptibility testing, if this treatment option is required. Whole genome sequencing appears to be a useful aid in GBS surveillance, with the potential to correlate findings to historical serotype data, MLST sequence types, and the presence of antimicrobial resistance and virulence genes. Other potential roles would include outbreak or transmission investigations, characterization of novel hypervirulent strains, and unexplored antimicrobial resistance mechanisms.

## Data Availability Statement

The datasets presented in this study can be found in online repositories. The names of the repository/repositories and accession number(s) can be found in the article/Supplementary Material.

## Author Contributions

SJ: project conception, data collection, laboratory work, synthesis of data, and writing of article. PN and MP: project supervision and proof-reading of article. LF: project supervision, laboratory work and supervision, analysis of data, and proof-reading of article. All authors contributed to the article and approved the submitted version.

## Funding

Funding was acquired through NSW Health Pathology trust fund.

## Conflict of Interest

The authors declare that the research was conducted in the absence of any commercial or financial relationships that could be construed as a potential conflict of interest.

## Publisher's Note

All claims expressed in this article are solely those of the authors and do not necessarily represent those of their affiliated organizations, or those of the publisher, the editors and the reviewers. Any product that may be evaluated in this article, or claim that may be made by its manufacturer, is not guaranteed or endorsed by the publisher.
