# Supplementary material for: Epidemiology, Antimicrobial Resistance, and Virulence Determinants of Group B Streptococcus in an Australian Setting
Source: Front Microbiol. 2022 Jun 14;13:839079. doi: 10.3389/fmicb.2022.839079 (PMC9238357; doi:10.3389/fmicb.2022.839079)
Supplement: Supplementary file 1 [file Data_Sheet_1.docx]

**Supplementary Materials – Appendix 1 and 3.**

**Appendix 1.** Antimicrobial resistance genes and virulence genes used in analysis of GBS isolates.

| **Antimicrobial resistance genes** | **Sequence length (bp)** | **Accession number** | **Other aliases** |
| --- | --- | --- | --- |
| Penicillin-binding protein *pbp1A* | 2,253 | 1013087 | SAG0298 |
| Penicillin-binding protein *pbp2A* | 2,322 | 1014877 | SAG2066 |
| Penicillin-binding protein *pbp1B* | 2,298 | 1012933 | SAG0159 |
| Penicillin-binding protein *pbp2B* | 2,046 | 1013569 | SAG0765 |
| Penicillin-binding protein *pbp2X* | 2,259 | 1013076 | SAG0287 |
| Beta-lactam resistance factor *fibA* | 1,212 | 1013312 | SAG0509 |
| Beta-lactam resistance factor *fibB* | 1,236 | 1013311 | SAG0508 |
| Ribosomal methylase *ermA* | 738 | 12611547 | SPSE_1800 |
| Ribosomal methylase *ermB* | 738 | 6479254 | SAG2253 |
| Ribosomal methylase *ermT* | 735 | 20467166 | ST1311_p2 |
| Macrolide efflux *mefA* | 1,182 | 1013014 | SAG2254 |
| Macrolide efflux *mefE* | 1,212 | 933922 | Spr0971 |
| Tetracycline resistance protein *tetM* | 1,920 | 1013727 | SAG0923 |
| DNA gyrase subunit A *gyrA* | 2,460 | 1013764 | SAG0960 |
| DNA gyrase subunit B *gyrB* | 1,953 | 1013427 | SAG0623 |
| DNA topoisomerase IV subunit A *parC* | 2,460 | 1013960 | SAG1153 |
| DNA topoisomerase IV subunit B *parE* | 1,962 | 1013961 | SAG1154 |
| Dihydrofolate reductase *folA* | 495 | 1014121 | SAG1314 |
| Chloramphenicol acetyltransferase *cat* | 639 | 1013717 | SAG0913 |
| **Virulence genes** | **Sequence length (bp)** | **Accession number** | **Other aliases** |
| Surface protein *rib* | 4,170 | 1013235 | SAG0433 |
| C5a peptidase *scpB* | 3,452 | 1014043 | SAG1236 |
| Hyaluronate lyase *hylB* | 3,246 | 3685697 | SAK_1284 |
| Fibronectin-binding protein *fbp* | 2,262 | 1013636 | SAG0832 |
| Beta hemolysin *cylE* | 2,004 | 1013473 | SAG0669 |
| Adherence protein A *pavA* | 1,656 | 1013997 | SAG1190 |
| Laminin-binding protein *lmb* | 921 | 1014041 | SAG1234 |
| CAMP factor *cfb* | 768 | 1014854 | SAG2043 |

**Appendix 3**. GBS isolate assembly quality data.

| **Isolate number** | **PubMLST ID** | **Number of contigs** | **Genome fraction (%)** | **Total length** | **Length of largest contig** | **LG50** | **NG50** | **LG75** | **NG75** |
| --- | --- | --- | --- | --- | --- | --- | --- | --- | --- |
| WOL-1-1 | [10586](https://pubmlst.org/bigsdb?page=info&db=pubmlst_sagalactiae_isolates&id=10586) | 87 | 87.36 | 2 064 042 | 294 278 | 6 | 115 497 | 14 | 52 569 |
| WOL-1-2 | [5699](https://pubmlst.org/bigsdb?page=info&db=pubmlst_sagalactiae_isolates&id=5699) | 57 | 87.75 | 2 073 377 | 547 097 | 4 | 119 394 | 9 | 67 830 |
| WOL-1-3 | [5700](https://pubmlst.org/bigsdb?page=info&db=pubmlst_sagalactiae_isolates&id=5700) | 129 | 87.17 | 2 051 921 | 146 348 | 15 | 50 405 | 31 | 23 646 |
| WOL-1-4 | [5748](https://pubmlst.org/bigsdb?page=info&db=pubmlst_sagalactiae_isolates&id=5748) | 58 | 86.63 | 2 074 018 | 355 457 | 4 | 153 305 | 10 | 67 844 |
| WOL-1-5 | [5701](https://pubmlst.org/bigsdb?page=info&db=pubmlst_sagalactiae_isolates&id=5701) | 10040 | 84.87 | 3 643 270 | 37 401 | 58 | 10 042 | 138 | 4 696 |
| WOL-1-6 | [5749](https://pubmlst.org/bigsdb?page=info&db=pubmlst_sagalactiae_isolates&id=5749) | 46 | 86.37 | 2 161 262 | 787 388 | 3 | 263 802 | 5 | 124 996 |
| WOL-1-7 | [5901](https://pubmlst.org/bigsdb?page=info&db=pubmlst_sagalactiae_isolates&id=5901) | 78 | 86.62 | 2 111 054 | 355 441 | 5 | 157 577 | 10 | 69 257 |
| WOL-1-9 | [5911](https://pubmlst.org/bigsdb?page=info&db=pubmlst_sagalactiae_isolates&id=5911) | 49 | 86.08 | 2 023 421 | 329 960 | 4 | 207 324 | 9 | 91 003 |
| WOL-1-10 | [10567](https://pubmlst.org/bigsdb?page=info&db=pubmlst_sagalactiae_isolates&id=10567) | 44 | 87.57 | 2 114 324 | 567 076 | 3 | 207 545 | 8 | 91 461 |
| WOL-1-13 | [5702](https://pubmlst.org/bigsdb?page=info&db=pubmlst_sagalactiae_isolates&id=5702) | 51 | 85.52 | 2 082 735 | 404 264 | 6 | 94 672 | 13 | 62 384 |
| WOL-1-14 | [5703](https://pubmlst.org/bigsdb?page=info&db=pubmlst_sagalactiae_isolates&id=5703) | 62 | 88.79 | 2 228 426 | 493 939 | 3 | 326 642 | 8 | 78 559 |
| WOL-1-15 | [5704](https://pubmlst.org/bigsdb?page=info&db=pubmlst_sagalactiae_isolates&id=5704) | 54 | 85.80 | 1 968 215 | 604 619 | 4 | 103 137 | 11 | 62 910 |
| WOL-1-16 | [5705](https://pubmlst.org/bigsdb?page=info&db=pubmlst_sagalactiae_isolates&id=5705) | 56 | 86.67 | 2 099 617 | 505 667 | 4 | 157 575 | 8 | 91 383 |
| WOL-1-17 | [5706](https://pubmlst.org/bigsdb?page=info&db=pubmlst_sagalactiae_isolates&id=5706) | 60 | 87.71 | 2 098 827 | 414 196 | 5 | 126 155 | 10 | 73 795 |
| WOL-1-18 | [10568](https://pubmlst.org/bigsdb?page=info&db=pubmlst_sagalactiae_isolates&id=10568) | 56 | 87.53 | 2 114 278 | 566 317 | 4 | 119 638 | 10 | 67 844 |
| WOL-1-19 | [5707](https://pubmlst.org/bigsdb?page=info&db=pubmlst_sagalactiae_isolates&id=5707) | 50 | 88.08 | 2 146 252 | 894 148 | 2 | 770 937 | 2 | 770 937 |
| WOL-1-21 | [5686](https://pubmlst.org/bigsdb?page=info&db=pubmlst_sagalactiae_isolates&id=5686) | 74 | 86.50 | 2 119 238 | 286 564 | 5 | 138 153 | 11 | 64 037 |
| WOL-1-22 | [5687](https://pubmlst.org/bigsdb?page=info&db=pubmlst_sagalactiae_isolates&id=5687) | 89 | 87.68 | 2 134 119 | 499 645 | 4 | 161 370 | 9 | 82 524 |
| WOL-1-23 | [5688](https://pubmlst.org/bigsdb?page=info&db=pubmlst_sagalactiae_isolates&id=5688) | 82 | 86.69 | 2 118 418 | 660 680 | 2 | 455 144 | 7 | 91 040 |
| WOL-1-24 | [5689](https://pubmlst.org/bigsdb?page=info&db=pubmlst_sagalactiae_isolates&id=5689) | 147 | 86.39 | 2 037 105 | 101 755 | 18 | 40 506 | 36 | 19 326 |
| WOL-1-25 | [5690](https://pubmlst.org/bigsdb?page=info&db=pubmlst_sagalactiae_isolates&id=5690) | 161 | 87.03 | 2 109 745 | 139 994 | 15 | 55 140 | 27 | 32 537 |
| WOL-1-26 | [5691](https://pubmlst.org/bigsdb?page=info&db=pubmlst_sagalactiae_isolates&id=5691) | 174 | 86.11 | 2 012 428 | 133 694 | 16 | 42 382 | 33 | 21 594 |
| WOL-1-29 | [6126](https://pubmlst.org/bigsdb?page=info&db=pubmlst_sagalactiae_isolates&id=6126) | 40 | 87.68 | 2 126 873 | 493 671 | 5 | 119 396 | 10 | 67 723 |
| WOL-1-31 | [5892](https://pubmlst.org/bigsdb?page=info&db=pubmlst_sagalactiae_isolates&id=5892) | 41 | 88.07 | 2 033 533 | 464 465 | 3 | 200 056 | 10 | 63 567 |
| WOL-1-32 | [5893](https://pubmlst.org/bigsdb?page=info&db=pubmlst_sagalactiae_isolates&id=5893) | 48 | 87.45 | 2 182 955 | 499 648 | 4 | 131 091 | 9 | 91 441 |
| WOL-1-33 | [5894](https://pubmlst.org/bigsdb?page=info&db=pubmlst_sagalactiae_isolates&id=5894) | 54 | 87.72 | 2 112 273 | 433 796 | 4 | 157 575 | 8 | 91 806 |
| WOL-1-34 | [5708](https://pubmlst.org/bigsdb?page=info&db=pubmlst_sagalactiae_isolates&id=5708) | 84 | 88.15 | 2 178 435 | 697 847 | 3 | 178 361 | 7 | 85 181 |
| WOL-1-35 | [6127](https://pubmlst.org/bigsdb?page=info&db=pubmlst_sagalactiae_isolates&id=6127) | 60 | 87.33 | 2 139 506 | 275 182 | 6 | 133 083 | 12 | 83 159 |
| WOL-1-36 | [5895](https://pubmlst.org/bigsdb?page=info&db=pubmlst_sagalactiae_isolates&id=5895) | 76 | 85.63 | 2 215 521 | 739 566 | 2 | 655 688 | 4 | 133 071 |
| WOL-1-37 | [10569](https://pubmlst.org/bigsdb?page=info&db=pubmlst_sagalactiae_isolates&id=10569) | 66 | 86.75 | 2 045 794 | 613 476 | 3 | 124 300 | 9 | 65 479 |
| WOL-1-38 | [5750](https://pubmlst.org/bigsdb?page=info&db=pubmlst_sagalactiae_isolates&id=5750) | 81 | 87.39 | 2 079 107 | 478 379 | 3 | 253 097 | 8 | 76 678 |
| WOL-1-39 | [5709](https://pubmlst.org/bigsdb?page=info&db=pubmlst_sagalactiae_isolates&id=5709) | 169 | 86.24 | 2 042 607 | 325 772 | 6 | 87 593 | 13 | 62 518 |
| WOL-1-40 | [5710](https://pubmlst.org/bigsdb?page=info&db=pubmlst_sagalactiae_isolates&id=5710) | 163 | 85.81 | 2 009 235 | 102 417 | 19 | 40 509 | 36 | 21 094 |
| WOL-1-42 | [5896](https://pubmlst.org/bigsdb?page=info&db=pubmlst_sagalactiae_isolates&id=5896) | 126 | 85.61 | 2 224 001 | 711 110 | 2 | 523 571 | 5 | 127 001 |
| WOL-1-43 | [5711](https://pubmlst.org/bigsdb?page=info&db=pubmlst_sagalactiae_isolates&id=5711) | 48 | 85.83 | 1 985 053 | 691 586 | 2 | 538 162 | 6 | 80 685 |
| WOL-1-44 | [6128](https://pubmlst.org/bigsdb?page=info&db=pubmlst_sagalactiae_isolates&id=6128) | 80 | 90.02 | 2 138 474 | 247 726 | 8 | 89 698 | 17 | 40 441 |
| WOL-1-46 | [6129](https://pubmlst.org/bigsdb?page=info&db=pubmlst_sagalactiae_isolates&id=6129) | 66 | 86.78 | 2 097 408 | 295 721 | 6 | 121 960 | 13 | 48 965 |
| WOL-1-47 | [5751](https://pubmlst.org/bigsdb?page=info&db=pubmlst_sagalactiae_isolates&id=5751) | 70 | 92.39 | 2 154 459 | 203 635 | 8 | 96 293 | 15 | 63 701 |
| WOL-1-48 | [5897](https://pubmlst.org/bigsdb?page=info&db=pubmlst_sagalactiae_isolates&id=5897) | 67 | 86.43 | 2 060 490 | 317 387 | 7 | 98 638 | 14 | 59 783 |
| WOL-1-49 | [10574](https://pubmlst.org/bigsdb?page=info&db=pubmlst_sagalactiae_isolates&id=10574) | 120 | 92.39 | 2 232 422 | 339 255 | 6 | 103 307 | 14 | 61 346 |
| WOL-1-50 | [5752](https://pubmlst.org/bigsdb?page=info&db=pubmlst_sagalactiae_isolates&id=5752) | 44 | 87.00 | 2 125 613 | 520 822 | 4 | 213 192 | 7 | 114 614 |
| WOL-1-51 | [5753](https://pubmlst.org/bigsdb?page=info&db=pubmlst_sagalactiae_isolates&id=5753) | 75 | 86.32 | 2 112 537 | 373 090 | 4 | 176 401 | 9 | 67 205 |
| WOL-1-55 | [6113](https://pubmlst.org/bigsdb?page=info&db=pubmlst_sagalactiae_isolates&id=6113) | 78 | 88.56 | 2 126 764 | 457 333 | 4 | 170 341 | 9 | 67 791 |
| WOL-1-57 | [5898](https://pubmlst.org/bigsdb?page=info&db=pubmlst_sagalactiae_isolates&id=5898) | 93 | 90.03 | 2 143 194 | 247 551 | 8 | 69 676 | 18 | 41 428 |
| WOL-1-59 | [5712](https://pubmlst.org/bigsdb?page=info&db=pubmlst_sagalactiae_isolates&id=5712) | 59 | 87.30 | 2 144 922 | 404 204 | 4 | 178 299 | 9 | 83 855 |
| WOL-1-60 | [5754](https://pubmlst.org/bigsdb?page=info&db=pubmlst_sagalactiae_isolates&id=5754) | 49 | 88.49 | 2 137 505 | 447 122 | 4 | 191 797 | 8 | 79 957 |
| WOL-1-61 | [5713](https://pubmlst.org/bigsdb?page=info&db=pubmlst_sagalactiae_isolates&id=5713) | 82 | 84.83 | 2 033 328 | 260 801 | 8 | 91 964 | 15 | 54 174 |
| WOL-1-62 | [5714](https://pubmlst.org/bigsdb?page=info&db=pubmlst_sagalactiae_isolates&id=5714) | 175 | 86.01 | 2 025 397 | 288 745 | 6 | 99 079 | 13 | 52 382 |
| WOL-1-64 | [5899](https://pubmlst.org/bigsdb?page=info&db=pubmlst_sagalactiae_isolates&id=5899) | 72 | 85.58 | 2 092 151 | 475 051 | 5 | 126 863 | 11 | 63 248 |
| WOL-1-65 | [5715](https://pubmlst.org/bigsdb?page=info&db=pubmlst_sagalactiae_isolates&id=5715) | 224 | 91.76 | 2 174 745 | 258 315 | 8 | 88 495 | 18 | 37 807 |
| WOL-1-67 | [5716](https://pubmlst.org/bigsdb?page=info&db=pubmlst_sagalactiae_isolates&id=5716) | 60 | 85.50 | 2 011 386 | 489 600 | 3 | 143 516 | 8 | 86 225 |
| WOL-1-68 | [5900](https://pubmlst.org/bigsdb?page=info&db=pubmlst_sagalactiae_isolates&id=5900) | 93 | 86.52 | 2 126 102 | 432 064 | 5 | 104 834 | 12 | 58 951 |
| WOL-1-69 | [10570](https://pubmlst.org/bigsdb?page=info&db=pubmlst_sagalactiae_isolates&id=10570) | 61 | 86.64 | 2 073 543 | 361 266 | 4 | 182 769 | 10 | 63 023 |
| WOL-1-71 | [5902](https://pubmlst.org/bigsdb?page=info&db=pubmlst_sagalactiae_isolates&id=5902) | 34 | 88.37 | 2 114 329 | 849 909 | 2 | 710 485 | 3 | 133 019 |
| WOL-1-72 | [5903](https://pubmlst.org/bigsdb?page=info&db=pubmlst_sagalactiae_isolates&id=5903) | 74 | 91.02 | 2 141 741 | 381 671 | 7 | 102 900 | 14 | 62 836 |
| WOL-1-73 | [5904](https://pubmlst.org/bigsdb?page=info&db=pubmlst_sagalactiae_isolates&id=5904) | 47 | 88.16 | 2 168 313 | 491 108 | 4 | 178 538 | 9 | 85 180 |
| WOL-1-74 | [5905](https://pubmlst.org/bigsdb?page=info&db=pubmlst_sagalactiae_isolates&id=5905) | 86 | 88.00 | 2 030 965 | 351 931 | 5 | 103 038 | 11 | 63 057 |
| WOL-1-75 | [5906](https://pubmlst.org/bigsdb?page=info&db=pubmlst_sagalactiae_isolates&id=5906) | 45 | 87.31 | 2 067 568 | 682 182 | 2 | 577 035 | 5 | 91 378 |
| WOL-1-77 | [5907](https://pubmlst.org/bigsdb?page=info&db=pubmlst_sagalactiae_isolates&id=5907) | 50 | 87.49 | 2 121 768 | 437 941 | 4 | 172 909 | 8 | 91 762 |
| WOL-1-78 | [5908](https://pubmlst.org/bigsdb?page=info&db=pubmlst_sagalactiae_isolates&id=5908) | 53 | 85.58 | 2 056 479 | 404 268 | 4 | 134 047 | 11 | 59 782 |
| WOL-1-79 | [5909](https://pubmlst.org/bigsdb?page=info&db=pubmlst_sagalactiae_isolates&id=5909) | 71 | 90.97 | 2 137 496 | 275 240 | 5 | 187 634 | 11 | 72 927 |
| WOL-1-80 | [5755](https://pubmlst.org/bigsdb?page=info&db=pubmlst_sagalactiae_isolates&id=5755) | 103 | 87.59 | 2 084 374 | 652 327 | 4 | 105 694 | 10 | 67 692 |
| WOL-1-81 | [5910](https://pubmlst.org/bigsdb?page=info&db=pubmlst_sagalactiae_isolates&id=5910) | 53 | 86.7 | 2 023 538 | 650 948 | 3 | 215 708 | 8 | 63 696 |
| WOL-2-2 | [5916](https://pubmlst.org/bigsdb?page=info&db=pubmlst_sagalactiae_isolates&id=5916) | 65 | 85.63 | 2 027 648 | 384 289 | 4 | 186 953 | 10 | 67 698 |
| WOL-2-3 | [6114](https://pubmlst.org/bigsdb?page=info&db=pubmlst_sagalactiae_isolates&id=6114) | 50 | 87.20 | 2 088 637 | 369 421 | 4 | 198 372 | 9 | 77 195 |
| WOL-2-4 | [10587](https://pubmlst.org/bigsdb?page=info&db=pubmlst_sagalactiae_isolates&id=10587) | 63 | 86.54 | 2 032 243 | 361 264 | 4 | 244 499 | 7 | 91 813 |
| WOL-2-5 | [6130](https://pubmlst.org/bigsdb?page=info&db=pubmlst_sagalactiae_isolates&id=6130) | 137 | 86.54 | 2 031 775 | 277 101 | 6 | 141 728 | 12 | 62 552 |
| WOL-2-6 | [6115](https://pubmlst.org/bigsdb?page=info&db=pubmlst_sagalactiae_isolates&id=6115) | 60 | 88.62 | 2 146 094 | 322 796 | 6 | 113 940 | 12 | 75 905 |
| WOL-2-7 | [6116](https://pubmlst.org/bigsdb?page=info&db=pubmlst_sagalactiae_isolates&id=6116) | 49 | 85.78 | 1 967 379 | 604 924 | 3 | 124 306 | 9 | 63 707 |
| WOL-2-8 | [5924](https://pubmlst.org/bigsdb?page=info&db=pubmlst_sagalactiae_isolates&id=5924) | 111 | 88.08 | 2 059 857 | 348 038 | 5 | 132 838 | 11 | 74 301 |
| WOL-2-10 | [5912](https://pubmlst.org/bigsdb?page=info&db=pubmlst_sagalactiae_isolates&id=5912) | 73 | 87.48 | 2 118 855 | 325 286 | 6 | 119 402 | 11 | 67 973 |
| WOL-2-11 | [6131](https://pubmlst.org/bigsdb?page=info&db=pubmlst_sagalactiae_isolates&id=6131) | 368 | 88.51 | 2 248 969 | 319 042 | 6 | 161 335 | 11 | 63 254 |
| WOL-2-12 | [6132](https://pubmlst.org/bigsdb?page=info&db=pubmlst_sagalactiae_isolates&id=6132) | 50 | 87.41 | 2 073 091 | 478 381 | 3 | 228 429 | 10 | 63 183 |
| WOL-2-14 | [5913](https://pubmlst.org/bigsdb?page=info&db=pubmlst_sagalactiae_isolates&id=5913) | 55 | 86.22 | 2 141 467 | 355 476 | 5 | 110 143 | 11 | 79 196 |
| WOL-2-16 | [5914](https://pubmlst.org/bigsdb?page=info&db=pubmlst_sagalactiae_isolates&id=5914) | 55 | 86.60 | 2 022 034 | 471 052 | 4 | 151 732 | 10 | 59 360 |
| WOL-2-18 | [5915](https://pubmlst.org/bigsdb?page=info&db=pubmlst_sagalactiae_isolates&id=5915) | 58 | 87.40 | 2 170 587 | 520 084 | 4 | 141 721 | 9 | 82 526 |
| WOL-2-19 | [5756](https://pubmlst.org/bigsdb?page=info&db=pubmlst_sagalactiae_isolates&id=5756) | 49 | 86.89 | 2 193 233 | 383 534 | 5 | 148 443 | 9 | 98 457 |
| WOL-2-20 | [5917](https://pubmlst.org/bigsdb?page=info&db=pubmlst_sagalactiae_isolates&id=5917) | 78 | 86.71 | 2 048 913 | 277 729 | 6 | 106 871 | 13 | 52 261 |
| WOL-2-21 | [5918](https://pubmlst.org/bigsdb?page=info&db=pubmlst_sagalactiae_isolates&id=5918) | 87 | 86.60 | 2 147 631 | 222 302 | 7 | 137 692 | 12 | 79 521 |
| WOL-2-22 | [6133](https://pubmlst.org/bigsdb?page=info&db=pubmlst_sagalactiae_isolates&id=6133) | 610 | 94.60 | 2 520 611 | 154 375 | 18 | 33 368 | 45 | 12 795 |
| WOL-2-23 | [6134](https://pubmlst.org/bigsdb?page=info&db=pubmlst_sagalactiae_isolates&id=6134) | 103 | 92.46 | 2 185 409 | 303 852 | 6 | 128 239 | 12 | 68 311 |
| WOL-2-24 | [5919](https://pubmlst.org/bigsdb?page=info&db=pubmlst_sagalactiae_isolates&id=5919) | 67 | 86.25 | 2 035 047 | 471 031 | 3 | 208 562 | 9 | 63 818 |
| WOL-2-25 | [5920](https://pubmlst.org/bigsdb?page=info&db=pubmlst_sagalactiae_isolates&id=5920) | 72 | 87.87 | 2 082 495 | 283 847 | 5 | 157 577 | 10 | 93 192 |
| WOL-2-27 | [5921](https://pubmlst.org/bigsdb?page=info&db=pubmlst_sagalactiae_isolates&id=5921) | 83 | 90.69 | 2 130 518 | 283 937 | 8 | 80 268 | 16 | 62 195 |
| WOL-2-30 | [5757](https://pubmlst.org/bigsdb?page=info&db=pubmlst_sagalactiae_isolates&id=5757) | 42 | 88.12 | 2 062 379 | 685 268 | 3 | 191 272 | 6 | 116 926 |
| WOL-2-31 | [5922](https://pubmlst.org/bigsdb?page=info&db=pubmlst_sagalactiae_isolates&id=5922) | 45 | 87.13 | 2 148 959 | 360 042 | 4 | 218 404 | 7 | 114 574 |
| WOL-2-32 | [10572](https://pubmlst.org/bigsdb?page=info&db=pubmlst_sagalactiae_isolates&id=10572) | 217 | 86.25 | 5 004 654 | 708 792 | 2 | 584 610 | 3 | 527 810 |
| WOL-2-34 | [6117](https://pubmlst.org/bigsdb?page=info&db=pubmlst_sagalactiae_isolates&id=6117) | 51 | 87.80 | 2 079 987 | 352 562 | 6 | 119 403 | 11 | 67 728 |
| WOL-2-36 | [10573](https://pubmlst.org/bigsdb?page=info&db=pubmlst_sagalactiae_isolates&id=10573) | 43 | 86.62 | 2 063 909 | 888 819 | 2 | 216 256 | 8 | 81 716 |
| WOL-2-37 | [5758](https://pubmlst.org/bigsdb?page=info&db=pubmlst_sagalactiae_isolates&id=5758) | 61 | 86.24 | 2 043 755 | 387 300 | 5 | 124 289 | 11 | 63 330 |
| WOL-2-38 | [6118](https://pubmlst.org/bigsdb?page=info&db=pubmlst_sagalactiae_isolates&id=6118) | 55 | 86.36 | 2 146 434 | 379 037 | 4 | 235 974 | 10 | 62 699 |
| WOL-2-39 | [5759](https://pubmlst.org/bigsdb?page=info&db=pubmlst_sagalactiae_isolates&id=5759) | 40 | 86.11 | 2 075 170 | 441 362 | 5 | 177 257 | 9 | 89 064 |
| WOL-2-40 | [5760](https://pubmlst.org/bigsdb?page=info&db=pubmlst_sagalactiae_isolates&id=5760) | 32 | 86.89 | 2 145 131 | 731 835 | 3 | 263 547 | 5 | 147 391 |
| WOL-2-41 | [5761](https://pubmlst.org/bigsdb?page=info&db=pubmlst_sagalactiae_isolates&id=5761) | 67 | 92.18 | 2 097 551 | 215 218 | 7 | 118 494 | 13 | 61 297 |
| WOL-2-42 | [6119](https://pubmlst.org/bigsdb?page=info&db=pubmlst_sagalactiae_isolates&id=6119) | 66 | 87.85 | 2 078 599 | 651 057 | 3 | 251 137 | 7 | 78 808 |
| WOL-2-43 | [5923](https://pubmlst.org/bigsdb?page=info&db=pubmlst_sagalactiae_isolates&id=5923) | 109 | 91.76 | 4 870 183 | 919 403 | 2 | 355 444 | 4 | 251 814 |
| WOL-2-50 | [6120](https://pubmlst.org/bigsdb?page=info&db=pubmlst_sagalactiae_isolates&id=6120) | 55 | 84.87 | 2 011 443 | 280 660 | 7 | 90 046 | 15 | 46 436 |
| WOL-2-51 | [6135](https://pubmlst.org/bigsdb?page=info&db=pubmlst_sagalactiae_isolates&id=6135) | 61 | 86.74 | 2 103 389 | 533 344 | 3 | 151 899 | 8 | 81 931 |
| WOL-2-53 | [6136](https://pubmlst.org/bigsdb?page=info&db=pubmlst_sagalactiae_isolates&id=6136) | 97 | 92.24 | 2 187 299 | 255 792 | 6 | 116 052 | 14 | 51 291 |
| WOL-2-54 | [5717](https://pubmlst.org/bigsdb?page=info&db=pubmlst_sagalactiae_isolates&id=5717) | 88 | 87.38 | 2 081 743 | 478 352 | 3 | 282 253 | 8 | 81 879 |

Note: Data obtained via alignment to AE009948 Reference genome. Genome fraction refers to the total number of aligned bases in the reference, divided by the genome size. LG50 refers to the minimum number of contigs that produce half (50%) of the bases of the reference genome (i.e. the number of contigs of length at least NG50). NG50 is the contig length such that using longer or equal length contigs produces half (50%) of the bases of the reference genome. LG75 and NG75 are similar terms referring to that covering 75% of the bases of the reference genome.
