# Supplementary figures and images for: Epidemiology, Antimicrobial Resistance, and Virulence Determinants of Group B Streptococcus in an Australian Setting
Source: Front Microbiol. 2022 Jun 14;13:839079. doi: 10.3389/fmicb.2022.839079 (PMC9238357; doi:10.3389/fmicb.2022.839079)

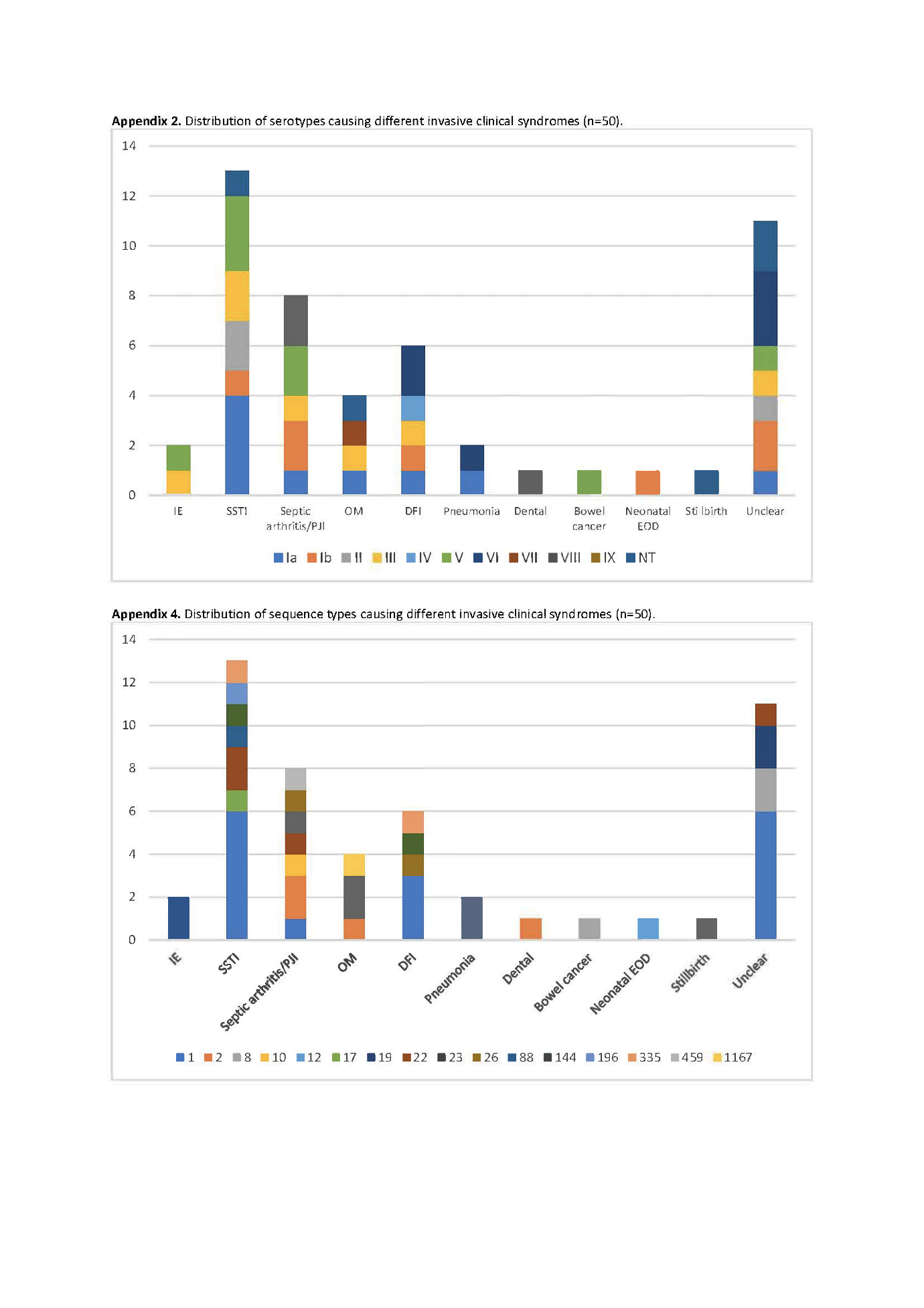

Supplement: Supplementary file 2 [file Data_Sheet_2.docx]
